# Outcomes of Definitive Chemoradiotherapy for Stage IVa (T4b vs. N4) Esophageal Squamous Cell Carcinoma Based on the Japanese Classification System: A Retrospective Single-Center Study

**DOI:** 10.3390/cancers13010008

**Published:** 2020-12-22

**Authors:** Yuki Wada, Akira Anbai, Noriko Takagi, Satoshi Kumagai, Eriko Okuyama, Hiroshi Nanjo, Yusuke Sato, Satoru Motoyama, Manabu Hashimoto

**Affiliations:** 1 Department of Radiology, Akita University Graduate School of Medicine, 1-1-1 Hondo, Akita 010-8545, Japan; anbai@doc.med.akita-u.ac.jp (A.A.); tnoriko@med.akita-u.ac.jp (N.T.); skuma@med.akita-u.ac.jp (S.K.); eri.lily@med.akita-u.ac.jp (E.O.); hashimms@med.akita-u.ac.jp (M.H.); 2 Division of Clinical Pathology, Akita University Hospital, 1-1-1 Hondo, Akita 010-8545, Japan; hnanjo@med.akita-u.ac.jp; 3 Esophageal Surgery, Akita University Hospital, 1-1-1 Hondo, Akita 010-8545, Japan; yusuke@doc.med.akita-u.ac.jp (Y.S.); motoyama@doc.med.akita-u.ac.jp (S.M.)

**Keywords:** esophageal squamous cell carcinoma, definitive chemoradiation therapy, external beam radiation therapy, locally advanced esophageal cancer

## Abstract

**Simple Summary:**

Definitive chemoradiotherapy is a standard treatment for stage IVa esophageal cancer, although it is unclear whether T/N factors affect treatment outcomes and whether we should modify chemoradiotherapy regimens based on T/N factors. This single-center retrospective study aimed to determine whether T/N factors affected progression patterns and overall survival after chemoradiotherapy for stage IVa esophageal squamous cell carcinoma. There were no significant differences between the T/N groups in terms of overall survival, progression-free survival, or progression patterns. Therefore, it may not be useful to modify chemoradiotherapy regimens based on T/N factors for patients with stage IVa squamous cell carcinoma.

**Abstract:**

The differences in prognoses or progression patterns between T4b non-N4 and non-T4b N4 esophageal squamous cell carcinoma post chemoradiotherapy (CRT) is unclear. This study compared the outcomes of CRT for stage IVa esophageal squamous cell carcinoma according to T/N factors. We retrospectively identified 66 patients with stage IVa esophageal squamous cell carcinoma who underwent definitive CRT at our center between January 2009 and March 2013. The treatment outcomes, i.e., progression patterns, prognostic factors, and toxicities based on version 5.0 of the National Cancer Institute Common Terminology Criteria for Adverse Events, were studied. The patients (56 men and 10 women) had a median age of 67 (range: 37–87) years. The T/N classifications were T4b non-N4 (28/66), non-T4b N4 (24/66), and T4b N4 (14/66). Objective response was achieved in 57 patients (86.4%, (95% confidence interval, 74.6–94.1%)). There were no significant differences between the T/N groups in terms of overall survival, progression-free survival, and progression pattern. We found no significant differences in prognoses or progression patterns among patients with T4b non-N4, non-T4b N4, and T4b N4 esophageal squamous cell carcinoma. Thus, it seems impractical to modify CRT regimens based on T/N factors.

## 1. Introduction

Esophageal cancer in Japan has a high mortality rate [1] and was reported in 2016–2017 as the 13th most common cancer [2]. Treatment options for esophageal cancer include endoscopic dissection, surgical resection, chemotherapy, and/or external beam radiotherapy, with specific combinations selected according to clinical stage and patient status. Especially in cases of advanced-stage disease, the treatment strategy should be carefully determined by a multidisciplinary team consisting of gastrointestinal physicians, esophageal surgeons, medical oncologists, diagnostic radiologists, and radiation oncologists. Definitive chemoradiotherapy (CRT) (50–60 Gy of radiation therapy and concurrent chemotherapy mainly with a combination of platina and fluorouracil) is generally provided with curative intent for locally advanced esophageal cancer when the patients’ condition is favorable [3].

In Japan, clinical staging of esophageal cancer can be determined based either on the Japanese Classification of Esophageal Cancer (edited by the Japanese Esophageal Society) [4] or the tumor, node, metastasis (TNM) classification of malignant tumors (edited by the Union For International Cancer Control (UICC)) [5]. While both systems assign the clinical stage based on the primary tumor (T factor), regional lymph node metastasis (N factor), and distant metastasis (M factor), there are several differences between them that are described in Table 1. In both systems, clinical stage IVa consists of locally advanced (T4) disease and/or widely spread lymph node metastasis (N4 in the Japanese system and N3 in the UICC system). The standard treatment for stage IVa disease involves CRT rather than surgical resection, although it is unclear whether there are differences in the failure patterns or survival rates according to specific T/N factors. Definitive CRT with the combination of full-dose radiation therapy and a full course of chemotherapy can sometimes induce treatment-related toxicities that cause delays in ongoing CRT or result in termination; thus, it would be favorable to reduce these toxicities by modifying the fields or doses of radiation therapy or the courses of chemotherapy. To consider this modification, it is important to clarify the recurrence pattern after CRT. Therefore, in this retrospective study, we aimed to evaluate the outcomes of CRT for stage IVa esophageal squamous cell carcinoma (based on the Japanese classification) and identify differences according to T/N factors that might permit modification of the radiotherapy and/or chemotherapy strategies.

## 2. Results

### 2.1. Patient Characteristics

The characteristics of the 66 patients (56 men and 10 women) included in this study are shown in Table 2. The median age at the start of CRT was 67 years (range: 37–87 years). We included only squamous cell carcinoma (*n* = 66; 100%) in this study due to the small number of other histological types at our institution. The T/N factor groups were T4bN0–3 (28 patients, 42.4%), T1–4aN4 (24 patients, 36.4%), and T4bN4 (14 patients, 21.2%). The most common chemotherapy regimen used was low-dose cisplatin (CDDP)/5-flurouracil (5-FU) (50 patients, 76%), followed by high-dose CDDP/5-FU (12 patients, 18%), nedaplatin (CDGP)/5-FU (two patients, 3%), 5-FU monotherapy (one patient with low renal function), and CDDP plus docetaxel (one patient). The median follow-up period was 12 months (range: 1–115 months).

### 2.2. Treatment Outcomes

**Figure 1:**
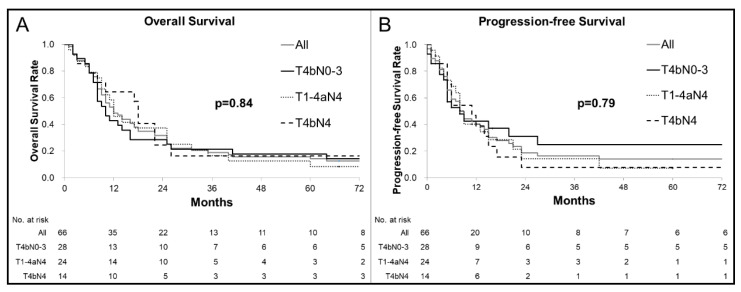
Kaplan–Meier curves for overall survival (OS) (**A**) and progression-free survival (PFS) (**B**): (**A**) Among all patients, the median OS was 13 months, with rates of 31.6% at 2 years and 14.2% at 5 years. There were no significant differences in OS between the patients with each T/N factor (*p* = 0.84). (**B**) Among all patients, the median PFS was 8 months and the 2-year PFS rate was 18.7%. There were no significant differences in PFS between the patients with each T/N factor (*p* = 0.79).

The best treatment responses were complete response (CR; 11 patients, 16.7% (95% confidence interval (95% CI), 1.26–51.1%)), partial response (PR; 46 patients, 69.7% (95% CI, 54.3–82.5%)), and stable disease (four patients, 6.1% (95% CI, 0.0–60.2%)). Objective response (CR + PR) was achieved in 57 patients (86.4% (95% CI, 74.6–94.1%)), although progressive disease (PD) was observed in five patients (7.6% (95% CI, 0.0–52.2%)) at first evaluation after starting CRT. Two patients whose esophageal cancers were reduced to an operable state had residual tumors after completing CRT and subsequently underwent salvage esophagectomy. Eighteen patients received further treatment for recurrent lesions, which included chemotherapy (13 patients), salvage CRT (two patients with lymph node recurrence outside the irradiated field), endoscopic submucosal dissection (two patients with local recurrence), and salvage surgery (one patient with locoregional lymph node recurrence). Figure 1 shows the progression-free survival (PFS) and overall survival (OS) curves. The median OS was 13 months, with OS rates of 31.6% at 2 years and 18.7% at 5 years. The median PFS was 8 months, and the 2-year PFS rate was 18.7%. A comparison of the T4bN0–3, T1–4aN4, and T4bN4 groups failed to detect significant differences in OS (*p* = 0.84) or PFS (*p* = 0.79).

### 2.3. Progression Patterns after CRT

The treatment failure patterns are summarized in Table 3. No significant differences were observed according to the T/N factors. No progression during the observation period was identified in 11 patients (39%) in the T4bN0–3 group, in six patients (26%) in the T1–4aN4 group, and in two patients (14%) in the T4bN4 group. Progression or recurrence in the T4bN0–3 group occurred inside the irradiated area with/without out-of-field recurrence (seven patients, 25%), outside the irradiated area with/without in-field recurrence (11 patients, 39%), and both inside and outside the irradiated area (one patient, 3.6%). In the T1–4aN4 group, progression or recurrence occurred inside the irradiated area with/without out-of-field recurrence (eight patients, 33%), outside the irradiated area with/without in-field recurrence (14 patients, 58%), and in both areas (four patients, 17%). In the T4bN4 group, progression or recurrence occurred inside the irradiated area with/without out-of-field recurrence (six patients, 43%) and outside the irradiated area with/without in-field recurrence (six patients, 43%).

### 2.4. Prognostic Factors

Table 4 shows the results of the univariate and multivariate analyses of OS. In univariate and multivariate analyses, no significant differences were observed, including in the T/N factors.

Table 5 shows the results of the univariate and multivariate analyses of PFS. In univariate and analyses, no significant differences were observed, including in the T/N factors.

### 2.5. Toxicities

Table 6 shows toxicities of grades 3–5 that occurred in 53 patients (80%). Late grade 5 adverse events were observed after CRT in five patients (8%) with the commonest adverse event being radiation pneumonitis (three patients at 4–15 months after completing CRT) who did not respond to steroids treatment. The lung parameters of radiation plan for theses patients were as follows: volumes of lungs irradiated with 20 Gy or more were 23%, 28%, and 40%, and mean lung doses were 12 Gy, 14 Gy, and 23 Gy, respectively. One patient’s lung irradiation dose was a little high; however, the other 2 patients’ lung irradiation doses were considered acceptable. One patient died of respiratory failure related to uncontrollable pleural effusion at 58 months after completing CRT, and another patient died of hemorrhagic shock related to esophageal hemorrhage from the irradiated area at 3 months after completing CRT. The most common grade 3 or more severe toxicities were hematological (49 patients, 74%), involving decreased white blood cell count (31 patients, 47%), anemia (20 patients, 30%), and decreased platelet count (11 patients, 17%). Grade 3 esophagitis was only observed in four patients. The definition of grade 3 esophagitis is “severely altered eating/swallowing; tube feeding, total parenteral nutrition, or hospitalization indicated” [6], although most patients already fulfill these criteria based on their locally advanced stage IVa esophageal cancer; this suggests that the rate of radiation esophagitis might have been underestimated.

## 3. Discussion

In this study, we compared the outcomes of CRT for stage IVa esophageal squamous cell carcinoma according to T/N factors and found a median OS of 13 months, a 2-year OS rate of 31.6%, and a CR rate of 16.7%; objective response (complete or partial response) was achieved in 86.4% of the patients. However, we did not manage to detect differences among the T4bN0–3, T1–4aN4, and T4bN4 groups in terms of their progression pattern, PFS, and OS.

Definitive CRT is standard treatment for stage IVa esophageal cancer, and there are many global reports on the outcomes from CRT in this setting. Most previous reports have used the UICC TNM classification of malignant tumors and considered T4 disease with/without M1 (lymph node) to be similar to the group that we evaluated in this study. Previous reports have indicated that the median OS in these cases is 5–14 months [7,8,9,10] with a 2-year OS rate of 31.5% [9] and a CR rate of 15% [9]. One study that used the Japanese classification revealed a median OS of 12.8 months, a 2-year OS rate of 35.1%, and a CR rate of 18.9% at stage IVa [11]. Similarly, the present study revealed a median OS of 13 months, a 2-year OS rate of 31.6%, and a CR rate of 16.7%, with objective response achieved in 57 patients (86%). These results are in accordance with those previously reported.

While stage IVa esophageal cancer includes locally advanced T4b; lymph node spread tendency N4; and T4bnon-N4, non-T4b N4, and T4b N4 disease, to our knowledge, no previous studies have examined their differences in outcomes or progression patterns. Previous reports showed that T4 of UICC is related to a poor CR rate of definitive CRT at approximately 25–32% [7,8,12] and that an increasing number of metastatic lymph nodes are related to poor OS rates in operative case studies [13,14]. Our results indicated that the T4bN0–3, T1–4aN4, and T4bN4 groups had similar outcomes in terms of progression pattern, PFS, and OS. Previous studies on definitive CRT for esophageal cancer, which included not only stage IV but also several T/N stages, have also indicated in-field recurrence and out-field recurrence being 40–50% and 43–50%, respectively [15,16,17]. These data suggest that both locoregional and distant recurrences commonly occur after definitive CRT for esophageal cancer. Thus, stage IVa esophageal cancer with either T4b or N4 requires both sufficient radiotherapy as local treatment and chemotherapy as a systemic treatment because progression can occur in and out of the irradiated field, regardless of T/N factors.

High toxicity when delivering sufficient radiotherapy and chemotherapy is a common complication. Concurrent CRT can cause significant toxicity [7,10,17], which may necessitate chemotherapy and/or radiotherapy delays or termination. For example, previous reports have indicated that only 50–67% of patients can complete their scheduled CRT at a planned dose of 50–60 Gy [18,19]. The present study found that 81% of the patients experienced grade 3 or more severe toxicities and that particularly 75% of the patients experienced grade 3 or more severe hematological toxicities, possibly leading to termination or delay in CRT. Previous studies that used nearly the same CRT regimen with elective nodal irradiation (ENI) setting have shown that grade 3 or more severe leukopenia, anemia, and thrombopenia were 73%, 30–33%, and 14–36%, respectively [12,17]. Therefore, while radiation and chemotherapy doses may need to be maintained to achieve optimal results, it is important to consider strategies that can reduce toxicity and enable patients to complete their planned treatment.

One conceivable strategy for managing toxicity is the modification of the radiation dose. However, in a previous study that compared CRT using the same chemotherapy regimen and a substantial radiation dose reduction (50 Gy vs. 30 Gy), inferior OS at the lower radiation dose was found [20]. The INT 0123 trial that included randomized patients with unresectable esophageal cancer who received CRT using radiation doses of 64.8 Gy or 50.4 Gy revealed that the higher dose did not provide superior locoregional control, leading to the broad adoption of 50.4 Gy as the standard radiation dose [21]. However, the introduction of newer radiotherapy techniques, such as image-guided radiation therapy, intensity modulated radiation therapy, and charged particle therapy, have led researchers to reconsider dose escalation in the French Concorde trial [22]. Unsatisfactory local control has been observed in Japan based on results from the JCOG9516 [9] and JCOG0303 trials [10], which has prompted our center to generally use a radiation dose of 60 Gy [23]. While further studies are needed to better understand the required radiation dose in this setting, it will be probably difficult to achieve satisfactory results with a dose of <50 Gy.

Another strategy for managing toxicity is to modify the irradiated field. For example, treatment may be limited to gross lesions while excluding the elective nodal area, which is known as involved field radiation therapy (IFRT). The elective nodal area has historically been targeted during CRT for esophageal cancer, based on the lymph node dissection pattern for definitive surgery in these cases, although it is unclear whether it is truly useful to target the elective nodal area in radiation therapy. Thus, it remains unclear whether ENI or IFRT should be preferred for esophageal cancer, although several retrospective studies have suggested that IFRT may provide advantages in terms of local control [24] and distant metastasis [24,25]. Those reports have also indicated that IFRT produced less toxicity than ENI, which suggests that patients may be more likely to complete their planned chemotherapy, thereby improving the treatment of distant metastasis. Moreover, if recurrence occurs in an area that was prophylactically irradiated using a dose of approximately 40 Gy, it would not be feasible to perform definitive radiotherapy at a dose of >50 Gy in the same area, which may preclude salvaged CRT for the recurrent lesion and may worsen local control and OS [26]. Neo-adjuvant CRT for esophageal cancer provides a pathological CR rate of only 23–68% when various chemotherapy regimens were combined with a radiation dose of approximately 40 Gy [27,28,29,30,31,32]. Thus, a prophylactic dose of 40 Gy for the ENI area in definitive CRT may be insufficient to achieve local control and might preclude adequate salvaged CRT if the patient experiences local lymph node recurrence. Although ENI may reduce regional nodal failure [17,33], these recent studies suggest that prophylactically expanding the irradiation field may not be prudent to reduce toxicities, especially hematological, and complete planned definitive CRT.

The present study has two important limitations. First, there are differences between the Japanese and UICC staging systems as well as country-specific differences in the histological types and accumulated doses. Second, the findings of this study are limited by the small retrospective single-center design. Thus, it is possible that our findings may not be replicated in other centers or regions. However, this study did not manage to detect differences among the T4bN0–3, T1–4aN4, and T4bN4 groups in terms of their progression pattern, PFS, and OS. Thus, our data suggest that sufficient dose of both radiation and chemotherapy is needed to improve the treatment outcome of stage IVa esophageal cancer regardless the T/N factors. However, it remains important to manage reduction in radiation-related toxicity, and it may be prudent to consider modifying the irradiated field (e.g., using IFRT) to improve the outcomes of definitive CRT for locally advanced esophageal cancer.

## 4. Materials and Methods

### 4.1. Patients

We identified 85 consecutive patients with clinical stage IVa esophageal cancer who were treated using radiotherapy, with or without other treatment modalities, between January 2009 and March 2013. Clinical staging was performed according to the Japanese Classification of Esophageal Cancer, 11th edition [4]. All patients’ stages and treatment strategies were determined by an institutional esophageal cancer board that consisted of esophageal surgeons, gastrointestinal physicians, medical oncologists, diagnostic radiologists, and radiation oncologists. All patients were judged as surgical operation was difficult or impossible, and the recommended therapy was definitive CRT.

Among the patients identified, we excluded a total of 19 patients: two who did not have squamous cell carcinoma (one adenocarcinoma and one verrucous carcinoma), four who received palliative radiotherapy limited to the primary esophageal lesions, five who did not receive chemotherapy because of their performance status or preference, and eight who became eligible for surgical resection due to tumor reduction before completing the definitive CRT at a dose of approximately 40 Gy. Therefore, this study included 66 patients who intended to receive definitive CRT with at least 50 Gy of radiation therapy and at least one course of systemic chemotherapy. We excluded non-squamous cell carcinoma because there was only one patient with adenocarcinoma and because verrucous carcinoma is a rare type of esophageal cancer. Therefore, we included only squamous cell carcinoma in this study in order to make the study cohort more homogenous. Their medical records were reviewed to collect data regarding laboratory findings, diagnostic images, radiotherapy planning, sex, age, pathological findings (including REG1 expression if available, which may predict the response to CRT) [34], PFS, OS, best treatment response, T/N factors, and adverse events.

### 4.2. Ethical Approval and Consent to Participate

The study’s retrospective protocol was approved by the Akita University Hospital institutional review board (no. 2138, approved on 21 February 2019). All methods were performed in accordance with the guidelines and regulations of the ethics board. Patient consent was not required based on the use of anonymized data.

### 4.3. Radiotherapy Planning

All radiotherapy plans were based on three-dimensional conformal radiotherapy. The target volumes were contoured using simulation computed tomography (CT) images, along with pretreatment diagnostic images (obtained using CT and 18F-fluoro-2-deoxy-D-glucose positron emission tomography (18FDG-PET)), gastrointestinal endoscopy findings, and clinical examinations, in accordance with the Japanese Society for Radiation Oncology guidelines [35]. The irradiated fields were determined using a combination of the target volume for the primary esophageal lesion and the target volume for the nodal area. The gross tumor volume for the primary esophageal lesion (GTVprimary) was defined based on its length, which was evaluated using radiography, CT, 18FDG-PET, or occasionally endoscopically placed clips. The clinical target volume for the primary lesion (CTVprimary) was defined as the GTVprimary plus an approximate craniocaudal margin of 3 cm for T1–4 cases (plus the periesophageal fat if clinically T4). The gross tumor volume of the clinically metastatic regional lymph nodes (GTVlymph) was defined based on the nodes’ volumes. The CTVlymph was generally equal to the GTVlymph without expansion. However, if extracapsular expansion of the lymph node was suspected, the CTVlymph was defined as the GTVlymph plus a 5-mm margin in all directions.

Although there is controversy regarding ENI [24,25,36,37], we generally included the elective nodal area with prophylactic intent. The elective nodal area varied slightly according to the primary lesion’s location, and CTVlympharea was generally determined according to the Japanese Society for Radiation Oncology guidelines [35]. For example, the cervical and abdominal regional lymph nodes are considered within the dissection area for surgical treatment. In the present study, 62 of 66 patients were treated with ENI including the prophylactic lymph node area expanded craniocaudally and the other 4 patients were treated with ENI including the lymph node area only near the primary lesion and lymph node metastases without expanding the prophylactic area craniocaudally. The planning target volume (PTV) considered PTV1 for the initial irradiation field and PTV2 for boost irradiation: PTV1 = (CTVprimary + CTVlymph + CTVlympharea) + 5–10 mm in all directions and PTV2 = (CTVprimary + CTVlymph) + 5–10 mm in all directions.

The dose reference point was defined at the iso-center. The radiation dose was fundamentally 60–61.2 Gy in 30–34 fractions over 6–7 weeks, and this was modified by radiation oncologists in charge according to the width of irradiation field, organs at risk included in irradiation area, patient condition, and so on. For example, 1.8 Gy per fraction was used instead of 2 Gy per fraction when the irradiation field was wide, and approximately total 50 Gy was used instead of approximately 60 Gy when the stomach was widely included in the irradiation field. These radiation doses were administered using high-energy 6-MV or 10-MV photons. Anterior–posterior opposing fields irradiation was initiated for PTV1 (approximately 40 Gy with range of 36.0–41.4 Gy in 20–23 fractions), which was followed by oblique portal irradiation fields for PTV2 (approximately 20 Gy) to spare the spinal cord. One patient stopped irradiation for PTV1 at 25.2 Gy and changed further irradiation areas for PTV2 due to a hematological adverse event, though 41.4 Gy for PTV1 was planned at the time of starting CRT.

### 4.4. Chemotherapy

Three chemotherapy regimens are generally used as part of CRT at our center: high-dose CDDP plus 5-FU, low-dose CDDP plus 5-FU, and nedaplatin (CDGP) plus 5-FU. These regimens were selected according to the patient’s age, performance status, liver/renal function, and other factors. The high-dose CDDP and 5-FU regimen involved an intravenous infusion of CDDP (80 mg/m^2^) on day 1 plus a continuous intravenous infusion of 5-FU (800 mg/m^2^) on days 1–5 [7]. The low-dose CDDP and 5-FU regimen involved an intravenous infusion of CDDP (40 mg/m^2^) on days 1 and 8 as well as continuous intravenous infusions of 5-FU (400 mg/m^2^) on days 1–5 and 8–12 [7]. The CDGP and 5-FU regimen involved an intravenous infusion of CDGP (90 mg/m^2^) on day 1 plus a continuous intravenous infusion of 5-FU (800 mg/m^2^) on days 1–5 [38]. The first cycle of chemotherapy started simultaneously with radiotherapy, with a plan to administer two chemotherapy cycles during radiotherapy. However, if grade 3 or worse hematological toxicities were observed, the second chemotherapy cycle was delayed until the toxicity resolved to grade 2 or better. Furthermore, if necessary, the drug doses were reduced by 20–30% according to age, performance status, liver/renal function, and toxicity severity. Continuous adjuvant chemotherapy was performed after the concurrent CRT for patients who experienced tumor response and were able to tolerate additional chemotherapy.

### 4.5. Outcomes

Treatment responses were evaluated using contrast-enhanced CT and gastrointestinal endoscopy based on version 1.1 of the Response Evaluation Criteria in Solid Tumors [39]. Any adverse events potentially related to CRT were identified using the patient’s medical records and reclassified based on version 5.0 of the National Cancer Institute Common Terminology Criteria for Adverse Events [6]. The interval for PFS was defined as the duration from the first day of CRT to the first day of identifying either progression or relapse or to the day of death without progression. The intervals for OS were defined as the duration from the first day of CRT to the day of death from any cause.

### 4.6. Statistical Analysis

The Kaplan–Meier method and log-rank test were used to compare the PFS and OS curves according to T4bN0–3, T1–T4aN4, and T4bN4 status. Relevant prognostic factors were identified using univariate analyses, and significant factors were included in the multivariate Cox proportional hazard model with stepwise backward regression. The T4bN0–3, T1–T4aN4, and T4bN4 groups were compared using the Pearson Χ^2^ contingency test with Yates correction for categorical variables and the Kruskal–Wallis test for continuous variables. The response rate was analyzed using the Clopper–Pearson confidence interval. A univariate Cox regression model was used to analyze the recurrence patterns of each T/N group to account for the time to progression. Differences were considered statistically significant at *p*-values of <0.05; all analyses were performed using BellCurve for Excel (version 3.20; Tokyo, Japan).

## 5. Conclusions

Similar to previous reports, the present study revealed that definitive CRT for stage IVa esophageal squamous cell carcinoma was associated with a median OS of 13 months and a 2-year OS rate of 32.1%. However, we failed to detect differences in the progression sites and survival rates between cases that involved T4b or N4 disease. Thus, these factors are likely not useful for modifying the chemoradiotherapy strategy in this setting, and further studies are needed to determine whether outcomes can be improved by modifying the prescribed dose and irradiated field (e.g., only targeting the gross lesions).

## Figures and Tables

**Table 1:** Japanese Classification of Esophageal Cancer and Union For International Cancer Control (UICC) tumor, node, metastasis (TNM) classification of malignant tumors.

Factors	Japanese Classification	UICC Classification
Clinical stage IVa	T4b any N M0 Any T N4 M0 Regardless of pathological type	Squamous cell carcinoma T4a N2 M0 T4b any N M0 Any T N3 M0 Adenocarcinoma T1–T4a N2 M0 T4b N0–2 M0 Any T N3 M0
T factors	T4: Tumor invades adjacent structures T4a: Pleura, pericardium, diaphragm, lung, thoracic duct, azygos vein, nerve T4b: Aorta, trachea, bronchus, pulmonary vein, pulmonary artery, vertebral body	T4: Tumor invades adjacent structures T4a: Tumor invades pleura, pericardium, azygos vein, diaphragm, or peritoneum T4b: Tumor invades other adjacent structures, such as the aorta, vertebral body, or trachea
N factors	Classified by site of metastatic regional lymph nodes N4: Metastasis to distant (Group 4) lymph nodes, regardless of whether any other group(s) of regional lymph nodes are involved	Classified by number of regional lymph nodes metastases N2: Metastasis in 3 to 6 regional lymph nodes N3: Metastasis in ≥7 regional lymph nodes

**Table 2:** Patient characteristics.

Factors	All Patients *n* = 66	T4bN0–3 *n* = 28	T1–4aN4 *n* = 24	T4bN4 *n* = 14
Age (years)				
Median (Range)	67 (37–87)	67.5 (37–80)	64 (44–87)	67.5 (54–86)
Sex				
Male	56	23	23	10
Female	10	5	1	4
Performance status (ECOG)				
0–1	55	22	20	13
2–4	11	6	4	1
Length of primary lesion				
≥5 cm	47	23	16	8
<5 cm	19	5	8	6
Eating *				
Possible	12	2	10	0
Partially possible	41	22	11	8
Impossible	13	4	3	6
Chemotherapy regimen				
LD-CDDP/5-FU	50	21	19	10
HD-CDDP/5-FU	12	6	2	4
CDGP/5-FU	2	0	2	0
5-FU	1	0	1	0
CDDP/DTX	1	1	0	0
Reg1 expression (*n* = 32)				
Positive	22	9	9	4
Negative	10	5	4	1

ECOG: Eastern Cooperative Oncology Group, LD-CDDP: low-dose cisplatin, 5-FU: 5-fluorouracil, HD-CDDP: high-dose cisplatin, CDGP: nedaplatin, DTX: docetaxel. * *p* < 0.01. The remaining *p*-values were nonsignificant.

**Table 3:** Univariable Cox regression analyses of progression patterns.

Groups	No Recurrence	In-Field Recurrence with/without Out-of-Field Recurrence		Out-of-field Recurrence with/without In-Field Recurrence		In- and Out-of-Field Recurrence
		Esophagus	Lymph node	Lymph node	Distant organ	
T4bN0–3 *n* = 28	11 (39%)	7 (25%)		11 (39%)		1 (3.6%)
		5 (18%)	3 (11%)	4 (14%)	8 (29%)	
T1–4aN4 *n* = 24	6 (25%)	8 (33%)		14 (58%)		4 (17%)
		5 (21%)	6 (25%)	7 (29%)	8 (33%)	
T4bN4 *n* = 14	2 (14%)	6 (43%)		6 (43%)		0 (0%)
		4 (29%)	2 (14%)	1 (7.1%)	5 (36%)	
HR (95% CI)		1.21 (0.62–2.38)	1.15 (0.54–2.46)	0.91 (0.43–1.93)	1.04 (0.60–1.80)	0.99 (0.31–3.12)

HR: hazard ratio, CI: confidence interval.

**Table 4:** Univariate and multivariate analyses of factors that are associated with overall survival.

Variables	Univariate *p*-Value	Multivariate *p*-Value
Age ≤ 70 years vs. >70 years	0.41	0.49
Initial hemoglobin > 10 g/dl vs. ≤10 g/dl	0.22	0.56
Nadir hemoglobin > 10 g/dl vs. ≤10 g/dl	0.59	0.62
Initial albumin > 3.5 g/dl vs. ≤3.5 g/dl	0.29	0.44
Nadir albumin > 3.5 g/dl vs. ≤3.5 g/dl	0.28	0.23
ECOG PS 0–1 vs. 2–4	0.11	0.11
T factor T4b vs. T1-4a	0.86	0.97
N factor N4 vs. N0–3	0.74	0.62
Length of primary lesion >5 cm vs. ≤5 cm	0.35	0.27
REG1 expression (*n* = 32) positive vs. negative	0.44	-*
Eating possible vs. partially possible vs. impossible	0.86	0.64

ECOG PS: Eastern Cooperative Oncology Group performance status. * REG1 expression is not included in the multivariate analysis due to the lack of data.

**Table 5:** Univariate and multivariate analyses of factors that are associated with progression-free survival.

Variables	Univariate *p*-Value	Multivariate *p*-Value
Age ≤ 70 years vs. >70 years	0.91	0.80
Initial hemoglobin > 10 g/dL vs. ≤10 g/dL	0.66	0.43
Nadir hemoglobin > 10 g/dL vs. ≤10 g/dL	0.19	0.19
Initial albumin > 3.5 g/dL vs. ≤3.5 g/dL	0.93	0.99
Nadir albumin > 3.5 g/dL vs. ≤3.5 g/dL	0.70	0.74
ECOG PS 0–1 vs. 2–4	0.59	0.48
T factor T4b vs. T1-4a	0.81	0.95
N factor N4 vs. N0–3	0.52	0.53
Length of primary lesion >5 cm vs. ≤5 cm	0.35	0.29
REG1 expression (*n* = 32) positive vs. negative	0.51	-*
Eating possible vs. partially possible vs. impossible	0.79	0.78

ECOG PS: Eastern Cooperative Oncology Group performance status. * REG1 expression is not included in the multivariate analysis due to the lack of data.

**Table 6:** Toxicities related to chemoradiation therapy (*n* = 66).

Toxicity	Grade 3	Grade 4	Grade 5
Decreased white blood cell count	31 (47%)	5 (8%)	0
Anemia	20 (30%)	1 (3%)	0
Decreased platelet count	7 (11%)	4 (6%)	0
Increased alanine or aspartate aminotransferase	2 (3%)	0	0
Hyponatremia	12 (18%)	1 (2%)	0
Hyperkalemia	4 (6%)	0	0
Hypokalemia	2 (3%)	1 (2%)	0
Esophagitis	4 (6%)	0	0
Anorexia	4 (6%)	0	0
Esophageal stenosis	2 (3%)	0	0
Malaise	1 (2%)	0	0
Radiation pneumonitis	5 (8%)	0	3 (5%)
Pleural effusion	0	0	1 (2%)
Febrile neutropenia	2 (3%)	0	0
Esophageal bleeding	0	0	1 (2%)
Esophageal fistula	2 (3%)	0	0

## Data Availability

“MDPI Research Data Policies” at https://www.mdpi.com/ethics.
